# Liberalization and Governance in the Geographical Distribution of Pharmacies in Spain

**DOI:** 10.3390/ijerph19010034

**Published:** 2021-12-21

**Authors:** Ángel Miramontes Carballada, Rubén C. Lois-González

**Affiliations:** Department of Geography, University of Santiago de Compostela, 15782 Santiago de Compostela, Spain; rubencamilo.lois@usc.es

**Keywords:** geography, health, pharmacy, territory, Spain

## Abstract

The Health system in Spain is considered one of the most valued public services by the population. In fact, during the first decade of the 21st century, Spain became a health tourism destination for people from central and northern European countries. In addition to the health infrastructure, the quality of medical and nursing care stands out. Something similar also happens with the Spanish pharmaceutical system. However, there are some characteristics that should be addressed from a Geography perspective. The pharmacies’ legal system does not consider them to be of public interest. One is when some of the main activities are the sale and distribution of medicines that are partially paid for by the Administration, that is, Social Security. In the same activity, the public function is combined with the private interests of the pharmacy owners to provide a balanced territorial service. One of the conclusions demonstrates how the borders that are sometimes created by the Administration are not always the most efficient in relation to the characteristics of the territory, nor do they provide the best service to the population and, therefore, create territorial imbalances within a country. To reach our conclusions, we carried out an exhaustive study of the pharmacy legislation in the EU and in Spain, as well as Geography of Health and theories of territorial location. We combined this information with statistics on the territorial characteristics of Spain. This allowed us to confirm the peculiarities that exist within the governance of the distribution of pharmacy offices in Spain.

## 1. Introduction

The first works into the Geography of Health that we know of, and with a similar structure to today’s works, date back to the first decades of the 20th century. These works focused on the spatial spread of diseases at different scales. Over the decades, the themes focused on issues related to inequalities, health policies, or the use and access to health services. Most of these studies were conducted in countries such as the UK, Germany, or the USA [1,2,3,4,5,6].

At the same time, it is also important to highlight that the Geography of Health has always integrated aspects related to demographic, social, economic, and environmental variables. It has become one of the areas of study in Geography with more multidimensional methodologies, borrowing from economists, sociologists, or medical doctors [7,8,9]. In any case, the consensus on the objective of the Geography of Health is that it focuses on generating knowledge about the analysis of the results of health policies, the organization of the population’s health services, and the development of the territory [10].

Hence, a geographer is a fundamental figure in the face of the frequent changes of location or privatization of health services on a global scale. The territory is a cause and an effect of health problems. Distance, socioeconomic structure, accessibility, or health services are basic concepts in today’s Geography. It is also necessary for more studies to be carried out on regional and local scales, evaluating the population’s access to health and the optimal location of new health services, as seen in works about the balance between the use of services and health [11,12,13], location optimization models [14,15,16], or the policies of privatization and the spatial reordering of the health services’ supply [17,18]. The diversity of methods is also wide, such as the evaluation of the distances travelled by users, consumer studies, or accessibility to the flow of patients [19,20]. Another type of variable is that related to demographic characteristics [21].

When studying the accessibility of a health service such as a pharmacy, supply and demand must be taken into account. Understanding the location and social characteristics of the services as the offer and the characteristics of the clients as the demand. Accessibility not only depends on the location, but on the ability to overcome certain barriers or poor access [22,23]. In fact, it is very important to know the real area of influence of a specific social and health service [24,25]. In any case, there is no uniform relationship between the increase in distance and the decrease in the use of health services such as a pharmacy. Sometimes the negative effect of distance can be counteracted by the size, quality of care, image, opening hours, etc. [26,27,28]. It is also very important to bear in mind that the distribution of premises or services in the territory, in addition to being governed by mercantilist and capitalist criteria, follows a Geography rationale when it comes to the location. Within this science there are spatial models that combine the economy and the territory, among which the traditional ones by VonThünen (1826), Weber (1909), Christaller (1933), Lösch (1940), or Krugman (1992) [29] stand out. The understanding of spatial dynamics in the economy serves not only to satisfy the needs of the clients but also as an instrument for territorial urban planning in cities or towns where there are centers of concentration and movement of goods and services.

This objective of this work was to carry out a territorial assessment of the service offered by pharmacies in Spain following some of the mentioned works on the Geography of Health, central locations, or migration of services, an objective influenced by both the characteristics of the territory (population, economy, etc.) and the existence of regulations that limit the relocation or opening of this type of establishment. This regulation is a controversial issue among pharmaceutical multinationals and the Spanish pharmacies’ professionals, who have slowed down attempts by the Spanish administration to generalize and liberalize the opening of new pharmacies on several occasions. In any case, we are also aware that liberalization must be carried out in a regulated way so as not to suffer from errors that have been seen in other areas of the EU, as was the case in Sweden, where the Swedish Medicines Agency decided in April 2015 to withdraw paracetamol from supermarkets after verifying that in the last 6 years (2009–2015), since it was allowed to be marketed from supermarkets and pharmacies, the number of cases of poisoning from its consumption increased by 40%. The authors of this work previously investigated what could be regarded as of significant interest for Applied Geography. In it, a real case study was carried out focusing on the territorial assessment for the relocation of a pharmacy from one place to another, alongside a private report the results of which were used as legal expert advice. This expert advice served to grant the relocation and also made recommendations for the legal reform of an obsolete Galician pharmacy law [30]. The knowledge acquired after carrying out the case study encouraged us to carry out this paper in which we went from the local and regional to the national scales, all Spain.

## 2. Materials and Methods

This research focused on two previous works: a report that we carried out as judicial experts and a scientific article, which because of this report, to this day, has created jurisprudence within the legislation of territorial organization of pharmacy offices in Galicia (Spain) [30]. The first is a contract signed between the owners of a pharmacy and a group of geographers to write a report that included a territorial assessment of the potentialities and weaknesses that the relocation of a pharmacy within the same municipality in Spain would entail. It was a report that would also be used as legal expert advice and subsequent defense by its authors in court. Finally, the document was submitted as an expert report and defended in the Supreme Court of Justice of Galicia (Spain) and was faced with opposition from other pharmacies (eight in total) to the pharmacy’s relocation. Finally, in the spring of 2020, the courts ruled in favor of the proposal defended and analyzed by the group of geographers, resulting in a new jurisprudence in the Galician and Spanish justice, unknown until now, since the relocation did not “comply” with the provisions of the Pharmacy Law of this territory. In addition, it should be noted that in the ruling the magistrates relied on multiple occasions on the content of the territorial expert advice report.

This report can be considered as the seed of a later scientific article and, therefore, the other antecedent of the present work. The article presented a complete study on the current state of the Geography of Health and, especially, regarding the location of services, as evidenced by the extensive references on this issue in the international context. Based on the analysis of a series of factors such as demand, supply, accessibility, etc., the inclusion of the report related to a whole series of territorial and sociodemographic repercussions that revealed the geographic interest of this article [30].

The method used to create this current article, an exhaustive bibliographic analysis, was undertaken and was divided into two thematic areas: on the one hand, the current pharmacies’ legislation and regulations in the EU and especially the case of Spain and, on the other, references relating to the Geography of Health, central locations, or migration of services. In addition, we analyzed the economy and the Galician, Spanish, and European population official databases. The official statistical data allowed us to complete the results of the work carried out on the territorial characterization of Spain. We completed the territorial analysis using cartographic techniques to arrive at our final reflections [31,32,33].

The methodology of this work was not innovative; however, what does deserve the effort and its publication is verifying how the contribution of territorial analyses such as the present one can help to complement the studies carried out from other sciences such as medicine, economics, or politics. Studying the characteristics of a territory, with the legislation that orders it and the reality of location of the service and demand (pharmacy-population) is one of the most frequent and applied methodologies that we can carry out from Geography: the relationship of a certain activity or a service with a territory.

The structure of the article is aimed at responding to the main objective of the work: to check if the Spanish pharmaceutical service is balanced and homogeneous within its territory. It also had two secondary objectives that support the main objective. Therefore, we made an approach to the study of the main characteristics of the pharmaceutical policy models at the European Union level and, secondly, we analyzed the different attempts to liberalize pharmacies in Spain. Finally, using the results of each of the secondary objectives, the discussion and conclusions were drawn up and the main objective of this article was answered, making a final reasoning about the efficiency and territorial homogeneity of pharmacies in Spain.

## 3. Results

### 3.1. The Governance of Pharmacies in the European Union and Liberalisation Attempts in Spain

The purpose of showing the European models, in addition to knowing what happens within the European Union, is because some of the attempts to modify the Spanish pharmaceutical system were initiated in the EU.

#### 3.1.1. Pharmacy Models in the European Union

In the European Union we find different systems of pharmaceutical regulation, from the most liberal to more interventionist ones. This is due to two reasons: firstly, because European legislation does not intervene in the planning of pharmacies and, secondly, because each country plans the distribution of pharmacies, their ownership, and the financing of medicines [34,35].

The European Commission, given the varied legislative typology present in the EU, is willing to unify pharmaceutical policies, even though the geographical distribution of pharmacies and the dispersal of medicines is the sovereignty of the EU member countries, as stated in article 26 of Directive 2005/36/EC of the European Parliament and of the Council of 7 September 2005. In fact, an example of their unifying effort occurred in 2006, when an expert ruling was drawn up in which they questioned the rules of territorial planning and ownership of pharmacies in Spain.

As regards territorial planning, the European Union considers that “a limitation in the number of pharmacies is not, as a quantitative measure, an adequate means to guarantee a good supply of medicines and may even be counterproductive in this regard” (Directive 2005/36/EC of the European Parliament and of the Council of 7 September 2005). Regarding ownership, the restriction that only pharmacists can own pharmacies and that it is prohibited to own or jointly own more than one pharmacy at the same time is considered excessive. These two peculiarities are considered non-negotiable within the Spanish pharmaceutical world. Below, we detail the current situation in the rest of the EU.

In the EU countries, four large pharmacy models can be found [34,35,36,37].

(1) Southern Europe model, Spain together with Italy. In this model, the State plans the opening of new pharmacies considering population criteria and the distance between them, and ownership is held exclusively by pharmacists (Articulo 1, Legge 8 Novembre 1991 n. 362. Norme di riordino del settore farmaceutico. Gazzetta Ufficiale n. 269, 16 Novembre 1991) (see Table 1).

In Spain, since Law 16/1997 on the regulation of pharmacy services, each Autonomous Region legislates the pharmaceutical management of its territory (Law 16/1997, of 25 April, on the regulation of pharmacies, BOE No. 100, 26 April 1997). Spain is the only country that does not have a single pharmaceutical regulation law for its entire national territory. The biggest difference between Spain and Italy is that the ownership is limited to one pharmacy per licensee, whereas, in Italy, each pharmacist is allowed to be the owner of up to four pharmacies simultaneously (Article 1, Legge 8 Novembre 1991 n. 362. Norme di riordino del settore farmaceutico. Gazzetta Ufficiale n. 269, 16 November 1991).

(2) Anglo-Saxon model. With a more liberal approach applied to the United Kingdom and the Netherlands, there are no restrictions on the ownership or opening of pharmacies. In any case, in the United Kingdom, pharmacies associated with the National Health Service (NHS) have a series of obligations towards the Department of Health (Arts. 6, 7 and 8, the NHS regulations of 2005, Statutory Instrument number 641, 10 March 2005). Here there is a low density of pharmacies linked to a poor geographical distribution, since there is a higher concentration in more populated areas and near health care centers, while access to medicines in rural areas has issues. To solve this situation, rural doctors have been authorized to dispense the prescriptions that they prescribe.

As for the ownership, it is not reserved only for pharmacists; rather, any person or company can own pharmacies, favoring the existence of pharmacy chains. The only requirement is that the pharmacy must be managed by a pharmacy graduate. It is important to note that there are no limitations on ownership for individuals or companies that may have financial interests in the pharmaceutical sector. In fact, pharmacy chains are often owned by wholesale drug distribution companies, as in the case of Alliance Unichem. This distributor has a high market share in the Netherlands and the United Kingdom, where it owns more than 15% of the pharmacies.

(3) Northern Europe model. Finland and Denmark have very low densities of pharmacies and ownership is an administrative license. In Sweden, they are owned by the state, although recently, it has undergone a big change, as we will see.

In Finland, Denmark, and Luxembourg, in theory, there is no legislation regulating the establishment of pharmacies. However, a new opening cannot be carried out without the authorization of the Government, which is responsible for limiting their number and setting their location according to demographic and geographic criteria.

Pharmaceutical planning policy is to ensure that there are pharmacies close to the places where drugs are prescribed and where there is a large flow of patients; hence, a high percentage of pharmacies are located in health centers. Pharmacists work for the State, from which they receive a salary stipulated by law (Article 59, Medicinal Act 395/1987, Decision of 10 April 1987, Ministry of Social Affairs and Health, Finland, amendments up to 296/2004 included).

In these countries, the ownership of a pharmacy is an administrative license that expires when the holder reaches retirement age or in the event of death; therefore, the relocation of the pharmacy is not allowed. When a pharmacy license becomes vacant, the health authorities call a merit contest for pharmacists in order to designate the most qualified person to manage the pharmacy. Only one pharmacy license per person is granted and the holder has the obligation to manage it directly.

(4) Central European Model. This is a mixed model applicable to France, Belgium, and Austria. These countries have adopted ideas from the Mediterranean and Anglo-Saxon models when it comes to pharmacy planning. Thus, in the case of France and Belgium, only population criteria are used, while, in Austria, population criteria and distances between pharmacies are applied. As far as ownership is concerned, for example, in Belgium there is no regulation regarding pharmacy ownership. Any natural or legal person can be the owner of one or more. The only condition is that it be managed by a pharmacist.

**Figure 1:**
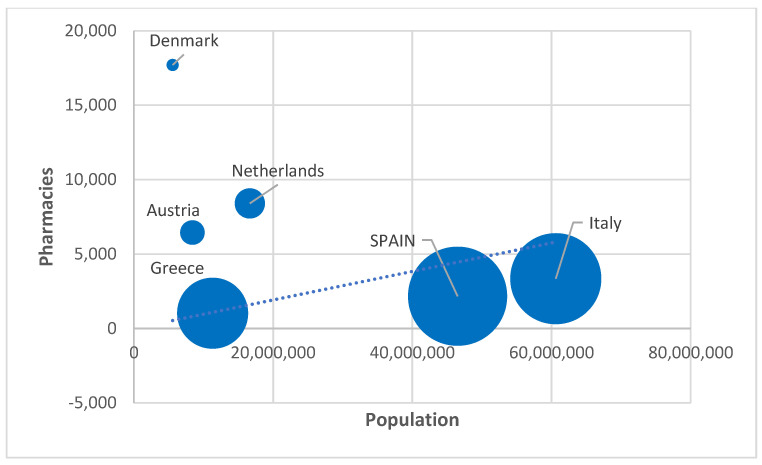
Representation of the population ratio per pharmacy in EU countries. Source: authors’ own work from [38].

For all this, in Figure 1 we summarize the characteristics of the geographical distribution of pharmacies, paying attention to the number of inhabitants, total pharmacies, and the ratio of inhabitant per pharmacy. The graph confirms the heterogeneity within Europe and the similarities between the countries of the South versus those of the North, as happens in many other socioeconomic and service variables.

In this type of analysis, there is the possibility that, at any moment, the government of a country may change its pharmacy management legislation (as happened in Sweden, with a radical change). In this country, all the pharmacies belonged to the State, which oversaw managing the entire network through an entity called Apoteket AB, which was responsible for setting up new pharmacies. The planning policy was to ensure that there were pharmacies close to the places where drugs were prescribed and with a large flow of patients. However, in 2009 the Swedish state began to draft a reform of the sector that was approved and based on the fact that anyone could have a pharmacy. One of the direct consequences was that the number of pharmacies was reduced, but their size increased to 10 to 15 staff members. This also resulted in the establishment of chains that brought together most of the country’s pharmacies. After these events, two questions arose: Will this happen in more European countries? Could this happen in Spain?

The EU has stated that the Spanish restrictions, where only pharmacists can own pharmacies and that it is prohibited to own or jointly own more than one pharmacy simultaneously, are excessive, as is the minimum number of inhabitants per pharmacy or the distance between them. It is endemic to the Spanish pharmaceutical world that the owner of a pharmacy, which is a public service in private hands, generates a profit for an individual of many hundreds of thousands of Euros a year (with an assured supply and a market competition “limited” by the Public Administration). Even in the Spanish case, unlike in other European countries, when the person who owns the pharmacy retires, he/she can resell the pharmacy for an estimated value of 10 years of net benefits, while getting the corresponding public pension, which will be the maximum amount due to their declared earnings.

#### 3.1.2. The Attempts to Liberalize Pharmacies in Spain

In recent decades, different proposals have been put forward to transform the pharmaceutical monopoly that exists in Spain and become more similar to other European systems. It is rather surprising that, despite the global economic crisis that began in 2008, Spanish pharmacies in the last 10 years have had an average annual turnover of just over 900,000 Euros, according to their Business Federation, with fluctuations that did not go beyond 2% or 3% with respect to any year in the last two decades. Even from 2015 to 2019, they increased by 11% on average. In fact, if we focus on the most current data, the average turnover of Spanish pharmacies in 2019 was more than 950,000 Euros. When grouping pharmacies by turnover, 20% exceeded 1.2 million Euros; 40%, 600,000; and another 40% did not reach that value. In any case, and as can be seen in Table 2, those in the lower range had an average that exceeded 475,000 Euros per year.

The statistics of the Spanish pharmacies base this clear increase on the higher sales of direct sale products (over the counter without a prescription such as creams, gels, etc.), while maintaining the sales of medicines.

Although it is an economic activity with values that are difficult to achieve in Spain for the self-employed, the situation brings greater criticism when the average number of employees in a Spanish pharmacy does not reach four, which equates to approximately one worker for every 225,000 Euros of sales. How much does a worker in a pharmacy earn? According to the Pharmacies Collective Labour Agreement, the most common categories are the associate pharmacist (who, as the name indicates, has a Higher Education Degree in Pharmacy, but is not an owner), who receives 14 pay checks a year of about 1800 Euros plus overtime. In the middle ground is the pharmacy technician (who has a Professional Training diploma) and receives 14 pay checks per year of 1220 Euros with on-call shifts, and, thirdly, the pharmacy assistant with 14 pay checks (two payrolls are paid in the months of June and December) of 1100 Euros with on-call shifts. Additionally, more questions may arise, such as: How much is the average salary in Spain? According to data from the Spanish Tax Agency in 2019, it was 1658 Euros per month. Therefore, a clear wage gap is detected between what the pharmacy owner and his/her employees and the rest of the workers in Spain earn.

These “advantageous” situations for pharmacy owners, compared to other workers, were the reasons behind some of the liberalization proposals that have been put forward in recent decades, and with the same negative result so far. One of the most determined attempts was when, in 2006, the European Union advised the socialist government of José Luis Rodríguez Zapatero to review the model with the aim of reducing macroeconomic imbalances in Spain and liberalizing certain heavily regulated professional sectors such as the pharmaceutical sector. However, it was never implemented due to pressure from the Professional Association of Spanish Pharmacists, supported by certain multinational laboratories.

Another of the processes to liberalize pharmacies and allow them to open freely took place at the end of 2012, but it did not go ahead on this occasion because, among other reasons, of the different points of view between the Ministry of Economy and Competitiveness (in favor) and the Ministry of Health (against).

The Ministry of Economy and Competitiveness proposed that any person, company, or large commercial chain could open a pharmacy with the sole condition of keeping the Pharmacy graduates as pharmacy managers, as is already the case in other European countries. The change was to be included in a draft bill for professional services, which would regulate other professions. The opposition of the Ministry of Health was blunt, with statements such as, “This model, in force in our country for decades, has guaranteed the professionalism of the pharmaceutical service and quality care at the service of the patient above other interests”, a spokesperson said. The current organization also ensures that anyone, both in rural and urban areas, can access medicines and health products. The dense network of pharmacies now allows 99.9% of the population to have one close to home. The General Council of Official Associations of Pharmacists spoke along the same lines, stating that “We have a close, accessible and quality system that all governments have maintained because its benefits citizens. It is also a health activity … “also, the measure would not generate savings, because what we dispense are medicines and the price is regulated. They are not consumer products but goods of social interest. Now, the citizen has the assurance that the pharmacist prioritizes public health before profit”, he added.

What is clear is that the debate has a territorial and social character, as is explained in the next section, where we analyze the regulations and the territorial distribution of pharmacies in Spain, in which Geography has much to contribute.

### 3.2. Presentation of Pharmaceutical Regulations and the Distribution of Pharmacies in Spain

In Spain, the opening of a new pharmacy is only permitted after obtaining the corresponding authorization, which is granted by the health department of each Autonomous Region. From each region, calls for the opening of pharmacies take place frequently, in accordance with the provisions of article 3 of Law 16/1997 on the Regulation of Pharmacy Services. Beyond these calls, the only way to set up a pharmacy is by purchasing an existing license or through inheritance. Currently, the average price of these licenses in Spain, with exceptions, are over 650,000 Euros, according to the data from companies that advise those interested in purchasing and selling pharmacy licenses [39,40].

In addition, the regulations also establish a series of requirements regarding the location of the pharmacy, both newly created and those that are to be relocated. Pharmacies are considered a public service and are highly regulated. In fact, pharmacies cannot be opened anywhere, but must be distributed throughout the Spanish territory in a homogeneous way to supply the entire population with medicines. In Spain, there are 22,028 pharmacies, which represent 2121 inhabitants per pharmacy (see Table 3). The range of the number of people per pharmacy on a regional scale is between 2643 people in the Basque Country and 1077 in Navarra (excluding the cases of Melilla or the Canary and Balearic Islands with values that exceed 3500 people). Those with the fewest inhabitants per pharmacy are Navarra, Castilla y León, and Extremadura. On the contrary, the high population level benefits the Canary Islands, the Basque Country, and Murcia.

This indicates that there is considerable heterogeneity between the economic profitability of a pharmacy and the service it provides to the population depending on the autonomous region in which they are located [34].

**Figure 2:**
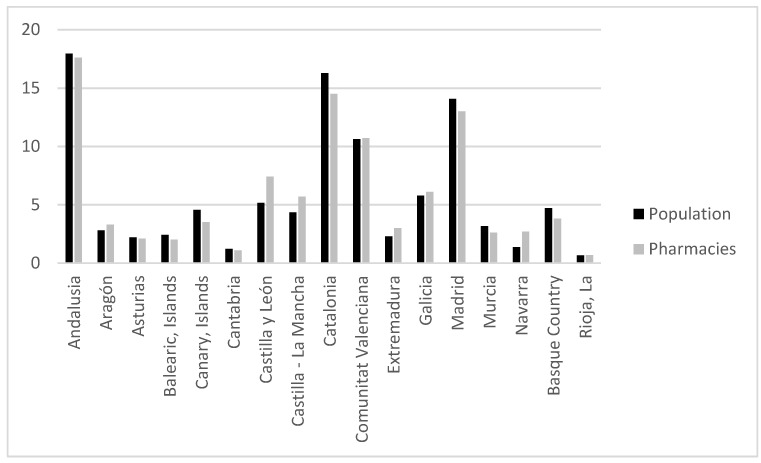
Percentage of the number of inhabitants and pharmacies of each of the autonomous regions over the total of Spain. Source: Authors’ own work based on data provided by the General Council of Official Associations of Pharmacists, 2019.

In fact, if we look at the number of inhabitants and the percentage of the number of pharmacies in each of the autonomous regions over the total of Spain (see Figure 2), the territorial homogeneity between the population and number of pharmacies that the regulation seeks to achieve is not equal for all Spain. The reason for this imbalance is found in the typology of population settlements and demographic density.

Regarding the territorial requirements that must be met to open a pharmacy, the following stand out:

(1) In each pharmaceutical area, only one pharmacy can be created per module of 2800 inhabitants.
(2) An additional pharmacy may only be opened if this proportion is exceeded, which will be created by the fraction greater than 2000 inhabitants.
(3) Each pharmacy must respect a minimum distance from existing pharmacies, which, as a rule, will be a minimum of 250 m.

Once the opening authorization has been granted, a series of additional requirements must be formalized. These are some of the most important: possess a full Pharmacy University Degree; Official Associations of Pharmacists (in Spain there are 52) holder’s registration number; deed of sale of the premises or lease, always within the areas delimited in the call; floorplan of the premises together with a report explaining the distribution of the different units that make up the pharmacy, location plan, or the declaration of possession of chemical products, as well as the devices and utensils required by the Spanish Pharmacopoeia, and the mandatory emergency medicines. This documentation is common for all Spain, and other, not so specific, reports, will also be requested such as safety or fire emergency reports.

In Galicia, a reform of the 1999 Law on Pharmaceutical Management, by current Law 3/2019, was recently approved. After 20 years of legal stagnation, no major changes were introduced, but there was an interest in the service reaching the entire population, such as allowing pharmacies to open branches in localities with no service. In fact, Section 4 of this Law includes the planning and territorial organization of new pharmacies. Once analyzed, some weaknesses or absence of territorial criteria or applied planning are evident. For example, in article 31 “Pharmaceutical areas”, the primary care units, which correspond to the municipal boundaries, are taken as the basis for planning. However, in turn, they create three pharmaceutical zones:

(1) Urban pharmaceutical areas: These correspond to municipalities with more than 30,000 inhabitants.
(2) Semi-urban pharmaceutical areas: These correspond to municipalities with a number of inhabitants between 10,000 and 30,000.
(3) Rural pharmaceutical areas: These correspond to municipalities with less than 10,000 inhabitants.

When examining this zoning approach, several questions and territorial weaknesses came up, such as: Do all the municipalities of Galicia with more than 30,000 inhabitants have the same territorial characteristics? Does the arrival of tourists have an impact? What about the workers who commute every day and are not registered as living in the destination? To answer all these questions within the recent Law, the Xunta de Galicia Council agreed to declare certain pharmaceutical areas as special.

Therefore, article 32 begins by establishing some modules to calculate the number of pharmacies that correspond to each area. These contents also raise a series of doubts, as discussed below.

(1) Urban pharmaceutical areas: one pharmacy for every 2800 registered inhabitants. Following the criteria applied throughout Spain, unless that proportion is exceeded by 1500 inhabitants, in which case a new pharmacy may be established if there has been a net population increase in the last 5 years.
(2) Semi-urban pharmaceutical areas: one pharmacy for every 2500 registered inhabitants, unless that proportion is exceeded by 1500 inhabitants, in which case a new pharmacy may be established as long as there has been a net population increase in the last 5 years.
(3) Rural pharmaceutical areas: one for every 2000 registered inhabitants, unless that proportion is exceeded by 1500, in which case a new pharmacy may be established as long as there has been a net population increase in the last 5 years.

Second, the article confirms that in each municipality there may be at least one pharmacy. This is A very positive and coherent measure to be able to offer this social health service to the entire population, but we found that it “contradicts” the criteria for delimiting the number of inhabitants. In Galicia, 43% of the municipalities have less than 2000 inhabitants [41].

Another determining variable within the territorial organization of pharmacies are distances. Article 33 “Distances between pharmacies” affects four aspects:

(1) The opening of a pharmacy, either new or as a result of relocation, may not be done at a distance less than 250 m from other pharmacies or from a public health care center open to the public or where it will be built as planned. This is the same distance that was already applied at a Spanish level.
(2) Exceptionally, in those pharmaceutical areas that have a single pharmacy, this minimum distance may not be required when there are reasons of general interest that justify it.
(3) The procedure, conditions, and criteria for measuring these distances is regulated.
(4) When a pharmacy is forced to temporarily move with an obligation to return, the minimum distance referred to in number 1 of this article is reduced to 125 m.

**Figure 3:**
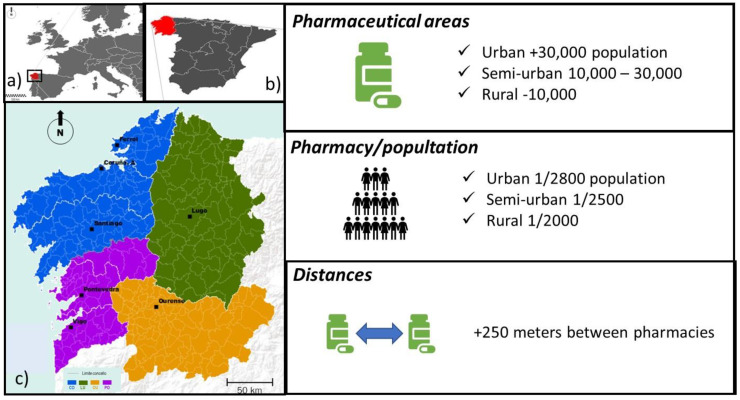
Geographical location and general characteristics that pharmacies in Galicia (Spain) must comply with. Geographic location of Galicia (**a**), in Europe (**b**), in Spain (**c**). Galicia is administratively divided into four provinces and 313 municipalities (by law, each municipality can have a pharmacy).

Therefore, we perceive a certain leniency in regard to the distance criteria, leaving “the door open” to the submission of general interest reasons or exceptions, which weaken the 250-m distance rule (see Figure 3).

Article 34 focuses on the Pharmaceutical Map and indicates that pharmaceutical planning criteria will be specified in accordance with the established regulatory procedure and will be approved by the Xunta de Galicia Council. A map that we considered was executed to a high level of competence. In any case, we detected a first weakness, since the pharmaceutical map groups all the pharmaceutical areas according to whether they are urban, semi-urban, or rural.

In addition, the map contains an annex with the new pharmacies and their specific territorial delimitations, indicating those that have a first-aid kit to cover the needs of places where, due to the restrictions or being special areas of difficult accessibility, it is not possible to open a new pharmacy.

Within the Law, the Administration “protects itself” by stating that the pharmaceutical map may be revised when significant alterations in the registry or other circumstances that lead to changes in the needs for pharmaceutical care are detected. It also specifies that the local administration or the official pharmacist associations may request a review of the pharmaceutical map before the competent council.

However, beyond the influence of population and distance variables, we highlight the need to study the characteristics of each territory regardless of scale when authorizing the opening or relocation of a public service with private ownership such as a pharmacy in Spain. Therefore, after applying a territorial analysis following Applied Geography criteria, we managed to get the competent health authorities of Spain and Galicia to authorize the relocation of a pharmacy, contrary to the current legislation in Galicia.

From our point of view, the criteria used were valid regardless of the territorial scale (see Table 4).

Having managed to get the Supreme Court of Justice of Galicia to contradict a previous ruling and reject the reports provided by the Regional Government and those submitted by a group of eight pharmacies, compared to the expert opinion carried out by a team of geographers, shows, in one more example, the significance that Geography and its decision-making capacity can have on the territory, as well as reaffirming that the correct analysis of the territory managed to modify the legislative criteria of the pharmaceutical regulations.

## 4. Discussion

We structured this section according to the proposed objectives and divided them into the three aspects that we studied in this paper: the territorial inequality of the pharmaceutical service, the liberalization of the sector, and the Spanish pharmacy as a historical example of public–private collaboration.

(1) In Spain, there is a very clear territorial imbalance within the pharmaceutical sector. Spanish legislation does not help at all to homogenize the pharmaceutical service, either at the Spanish level or internally within each of the 17 autonomous regions that have the most Health powers. It was proposed that, regardless of the existence of a basic legislation, which includes the criteria for planning pharmacies in Spain, it is necessary to legislate according to the characteristics of the territory to avoid provision imbalances such as those that exist today. Along the same lines, a Health pact is not necessary, but agreements for Health and the coordination in the provision of services and policies are. It is unreasonable to have 17 types of healthcare cards in Spain and some regions with a pharmacy for every 1000 inhabitants and in others one for every 4000 inhabitants. These are measures that could be implemented while the map of the pharmacies is readjusted according to the characteristics of the territory.
(2) Regarding the issue of liberalization, we understand the position of the General Council of the Official Associations of Pharmacists of Spain, which argued that the deregulation of the sector does not improve the situation in terms of social welfare and would harm the patient. Pharmacists affirm that they have a regulatory model for pharmacies with a per capita rate much higher than in countries where the sector is liberalized. In addition, they state that, when it is liberalized, pharmacy chains will play a bigger role, many times linked to the distribution of the sector where profit prevails over service. With liberalization, some pharmacies in certain current locations would disappear, centralizing the offer. In addition, Spanish pharmacists strive to convey that they are providing a health service to society and are not carrying out a commercial activity, the sale of a product.

In any case, and from our point of view, the group of pharmacists must also be aware that liberalization does not have to be total, as happened in other European countries. Although, after this study, we defend that it could be developed in a gradual and flexible way according to the characteristics of certain territorial areas. Liberalization would allow the highly speculative process on the final selling price of a pharmacy to be corrected, which normally reaches excessively high prices. 

Additionally, we do not see the move of medicine towards hospital dispensing as efficient. In terms of economic and social benefits, we affirm that the system does not work because the savings achieved by the Administration are carried by the patient (who has to pay for travel, take time off work, etc.). Let us remember that in Spain there are more than 22,000 dispensing points compared to 250 hospitals [42].

In short, despite agreeing with the Professional College of Pharmacists of Spain who oppose the liberalization of the pharmaceutical sector, the protectionism and the high profit margins by pharmacies in Spain are not acceptable either, as well as maintaining airtight regulations that shield the current pharmacy owners. The Spanish pharmaceutical map must be readjusted to the real characteristics of the territory: first, looking for a good pharmaceutical service for the whole of society and, second, with business characteristics closer to other health activities.

(3) Regardless of our negative interpretation of the management policy of the pharmaceutical map of Spain, we detected some positive elements of the service offered in Spain. In fact, different bodies acknowledge the Spanish pharmacy as a historical example of public–private collaboration [34]. As we have exposed on several occasions throughout this paper, Spanish pharmacies provide a public service that is managed by private interests.

## 5. Conclusions

Spain has one of the largest community pharmacy networks in Europe. However, in recent years it has been criticized by different sectors after various events. Thus, pharmaceutical spending has decreased significantly, even in per capita terms, with consumption values in 2017 like those of 2006 [34]. However, pharmaceutical care has maintained its activity.

Broadly speaking, we can affirm that the basic structures of the Spanish health system and its care model are correct. Another issue is that there is a debate on the role that each agent should play in the provision of services. We hope that when the economy recovers, there will be an increase in Public Health investment. One of the priorities of this investment will be to have health coverage closer to the standard parameters of neighboring countries, without falling into some of the mistakes of the past [42].

In any case, with this work, we consider that it is sufficiently clear that in Spain the map of pharmacies shows a clear territorial imbalance, which has somehow been improved over the last decades, but has not been consolidated. It has been rigorously drawn up by the administration and not according to the characteristics of the territory. Geographers believe that balance should be the key and that the territory can set the most appropriate criteria for the future.

## Figures and Tables

**Table 1:** Example of the main characteristics of the demographic and ownership criteria of pharmacies in EU countries.

	Ownership	Demographic Criteria
Spain	Limited to pharmacists. One pharmacy per licensee	Each Autonomous Region establishes its own criteria
Italy	Limited to pharmacists. Four pharmacies per licensee	In municipalities with a population: More than 12,500 inhabitants: One pharmacy/5000 inhabitants Less than 12,500 inhabitants: One pharmacy/4000 inhabitants
Netherlands	Not limited to pharmacists if managed by a pharmacist	Rural doctors prescribe and dispense their prescriptions. The closest pharmacy is more than 4.5 km away
Denmark	Limited to pharmacists and has to be managed by the owner	Administrative license that expires (retirement or death). The relocation of pharmacies is not allowed. It is accessed by calling a merit contest for pharmacists
Austria	Not limited to pharmacists	One pharmacy/5500 inhabitants The municipality must have a medical center

**Table 2:** Number of pharmacies by annual average billing values in 2019 in Spain.

Annual Turnover	Number of Pharmacies	Average Turnover (Euros/Year)
More than 1.2 million	4428	1,648,369
650,000 to 1,200,000	8880	913,432
Less than 650,000	8830	477,121 ^1^

^1^ Source: authors’ own work based on data from the Official College of Pharmacists of Spain and the Federation of Spanish Pharmaceutical Business Owners (FEFE in Spanish).

**Table 3:** Number of pharmacies by annual average billing values in 2019 in Spain.

	Population/Pharmacies
Melilla	3927
Ceuta	3548
Canary Islands	2792
Basque Country	2643
Murcia	2603
Balearic Islands	2577
Cantabria	2469
Catalonia	2378
Madrid	2299
Asturias	2260
Andalusia	2162
Spain Average	2121
Comunitat Valenciana	2105
Rioja, La	2024
Galicia	2010
Aragon	1783
Castilla-La Mancha	1602
Extremadura	1599
Castilla y Leon	1480
Navarra	1077

Source: Authors’ own work based on data provided by the General Council of Official Associations of Pharmacists, 2019.

**Table 4:** Basic criteria followed for the preparation of the expert report on the proposed relocation of a pharmacy in Galicia (Spain).

Criteria (Analysed)	Objective
Territorial legislation and regulations	Know the urban planning of the territory (partial, special plans, etc.).
Population structure	Mainly, take into account the strata of the aging population.
Economic structure	Detect the economic potentialities and weaknesses of the territory. The need for better care in vulnerable or poorer spaces.
Social culture	Know the idiosyncrasies and customs of the population. Introduce qualitative criteria for the consumption of the pharmaceutical products.
Population distribution	Focused on variables of density, distances, number of settlements, etc.
Provision of services and infrastructures	Linked to criteria of daily mobility or for working reasons.
Provision of health services and infrastructure	Characterisation of health services.
As a rule, work with percentages and trends on the space’s population and economic structure and not whole values.	

## Data Availability

This study does not report any data. The entire analysis was conducted using publicly available secondary data, and there are no data that are required to be made available.
